# DisVis: quantifying and visualizing accessible interaction space of distance-restrained biomolecular complexes

**DOI:** 10.1093/bioinformatics/btv333

**Published:** 2015-05-29

**Authors:** G.C.P. van Zundert, A.M.J.J. Bonvin

**Affiliations:** Bijvoet Center for Biomolecular Research, Faculty of Science - Chemistry, Utrecht University, Utrecht 3584CH, The Netherlands

## Abstract

**Summary:** We present DisVis, a Python package and command line tool to calculate the reduced accessible interaction space of distance-restrained binary protein complexes, allowing for direct visualization and quantification of the information content of the distance restraints. The approach is general and can also be used as a knowledge-based distance energy term in FFT-based docking directly during the sampling stage.

**Availability and implementation:** The source code with documentation is freely available from https://github.com/haddocking/disvis.

**Contact:** a.m.j.j.bonvin@uu.nl

**Supplementary information:** Supplementary data are available at *Bioinformatics* online.

## 1 Introduction

Structural characterization of protein complexes is of paramount importance for a fundamental understanding of cellular processes, and with major applications in rational drug design. As the quantity of experimentally determined complexes is only a fraction of their total predicted number, complementary computational techniques have been developed for predicting the structure of complexes from their components (Petrey and Honig, 2014; Rodrigues and Bonvin, 2014). Additional low-resolution information in the form of distance restraints can significantly benefit the modeling, with a variety of experimental methods providing such information, such as chemical cross-links detected by mass spectrometry (MS) (Rappsilber, 2011), and distance measurements from electron paramagnetic resonance (EPR) and FRET (Kalinin *et al.,* 2012).

When two biomolecules are known to interact and no high-resolution model is available, the structure of the complex can naively be any one state where the molecules are in contact. We define the accessible interaction space of the complex as the set of all these states. If a distance restraint is imposed on the complex, the accessible interaction space reduces, depending on the information content of the restraint. The interaction space is further reduced if multiple restraints are included. So far, however, no computational method has been reported that quantifies this reduction or allows to visualize this accessible interaction space.

To aid in this task, we have developed DisVis, a GPU-accelerated Python software package and command line tool (*disvis*) for quantifying and visualizing the accessible interaction space of distance-restrained binary complexes. *Disvis* takes as input two atomic structures and a file with distance restraints, and outputs the sum of complexes complying with a given number of restraints together with a density showing the maximum number of consistent restraints at every position in space. This indicates whether all data are consistent and can be combined without violations, and allows identification of false positives, quantification of the information content of the restraints and visualization of interesting regions in the interaction space. The method is generic and can easily be incorporated into existing Fast Fourier Transform (FFT)-accelerated docking programs as a distance-dependent energy function, allowing the ‘marriage made in heaven’ of direct sampling and scoring of FFT-generated docking poses (Vajda *et al*., 2013) at a small computational cost.

## 2 Methods

We discretely sample the accessible interaction space by treating the two biomolecules as rigid bodies and performing a 6 dimensional search over the three translational and three rotational degrees of freedom. We use FFT-techniques to accelerate the translational search using a 1 Å grid spacing (default). These have long been used in the docking field (Katchalski-Katzir *et al.,* 1992). One chain is fixed in space and considered the receptor molecule, while translational scans are performed for each rotation of the ligand molecule. Two atoms i and j are considered to be interacting if the distance, d, between them is $r_{\text{vdW}}<d\leq \;r_{\text{vdW}}+3Å$ (by default), where $r_{\text{vdW}}$ is the combined van der Waals radius of the two atoms $r_{\text{vdW}}^{i}+r_{\text{vdW}}^{j}$, and clashing if $d\leq \;r_{\text{vdW}}$. A conformation is deemed a complex if the volume of interaction is above- and the volume of clashes below threshold values (300 and 200Å^3^ by default, respectively).

After every translational scan, all conformations that comply with each restraint are determined. Next, *disvis* counts the number of complexes consistent with each number of restraints, as well as which restraints are violated. This is repeated until the rotational sampling density reaches a pre-set value (default 9.72°, 7416 orientations). During the rotational search, *disvis* stores the maximum number of consistent restraints found at every scanned position of the ligand’s center of mass, which ultimately results in a discrete ‘density’ map. The output thus consists of the sum of accessible complexes complying with each number of restraints, a percentage of how often each restraint is violated, and a discrete-valued density map.

We implemented DisVis in Python2.7, using the OpenCL framework to offload computations to the GPU. The code can be downloaded freely from https://github.com/haddocking/disvis together with documentation and examples.

## 3 Examples

**Fig. 1.**
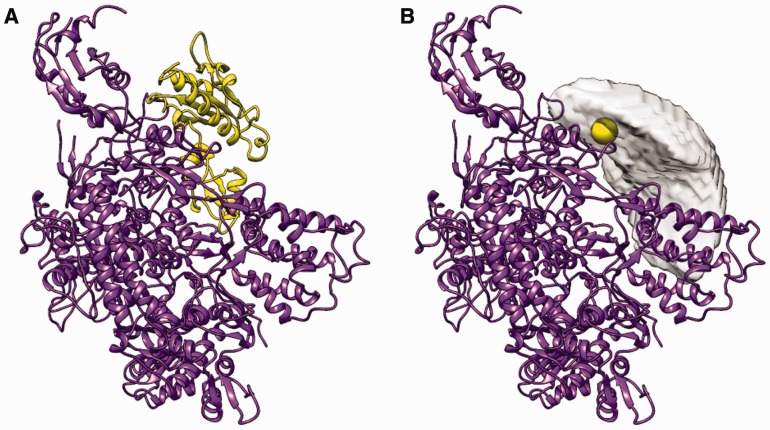
(**A**) The large subunit (purple) and the 27 kDa polypeptide (yellow) of the RNA polymerase II complex. (**B**) The large subunit and the reduced accessible interaction space of the 27 kDa polypeptide consistent with at least six cross-links in gray. The smooth yellow sphere represents the center of mass of the polypeptide

To illustrate the capabilities of *disvis,* we applied it on two systems, using MS cross-links data (see the Supplementary Information for details). A fine rotational search (5.27°, 53 256 orientations) was performed using default values. First, we investigated the accessible interaction space of two chains of the RNA polymerase II complex of S. cerevisiae (1WCM, chain A and E) for which 6 BS3 cross-links were available (Chen *et al.,* 2010; Kahraman *et al.*, 2013). The allowed distance was set between 0 and 30 Å (C_β_ – C_β_) for every restraint. Two false-positive restraints were added with a distance in the crystal structure of 35.7 (FP1) and 42.2 Å (FP2) to test whether they could be identified. Applying *disvis* shows that none of the 18.9 × 10^9^ complexes sampled are consistent with all 8 restraints, though a small number are conforming to 7 cross-links (9716 complexes). For the latter, only restraint FP2 is violated. The accessible interaction space consistent with at least 6 restraints is less than 0.03% of the full interaction space (Fig. 1). The density clearly indicates the position of the E-chain. Interestingly, both false-positive restraints are violated in 100% of the complexes consistent with at least six restraints; in contrast, the highest violation percentage of a correct cross-link is only 0.1%. Thus, a high-violation percentage is an indication of a false-positive restraint.

Second, we applied *disvis* on two proteins of the 26 S proteasome of S. pombe, PRE5 (O14250) and PUP2 (Q9UT97), with 7 cross-links available (Leitner *et al.*, 2014). The acceptable distances for the adipic acid dihydrazide (ADH) and zero-length (ZL) cross-links were set to 23 and 26 Å (C_α_ − C_α_), respectively, as 95% of distances found in a benchmark were shorter (Leitner *et al.,* 2014). The PRE5-PUP2 complex is significantly smaller than the previous example with the full interaction space consisting of 6.9 × 10^9^ complexes. Still, the accessible interaction space consistent with all 7 restraints is heavily reduced to less than 0.04% of the full interaction space. The accessible interaction space of the PUP2 chain with respect to PRE5 is overlapping with its center of mass deduced from a homology model (Supplementary Figure S1).

The computation for those two examples took 74 and 27 m on 16 AMD Opteron 6344 processors and 76 and 19 m on an NVIDIA GeForce GTX 680 GPU, respectively. However, by increasing the voxel spacing to 2 Å and using a coarser rotational search (9.72°, 7416 orientations) rather similar results can be obtained in only 19 and 8 m, respectively, on a single processor (cf. Supplementary Tables S2 and S4 for example). It should further be noted that the bulk of the time is spent on computing the FFTs and a negligible part on computing the consistent distance restraint space (Supplementary Table S11).

## 4 Conclusions

We have introduced DisVis, a Python package and command line tool to quantify and visualize the information content of distance restraints, and a powerful aid in detecting the presence of false-positive restraints. Our novel approach can be easily incorporated in FFT-accelerated docking programs, allowing the use of any form of distance-dependent energy function.

## Funding

This work was supported by the Dutch Foundation for Scientific Research (NWO) [ECHO grant no.711.011.009].

*Conflict of Interest*: none declared.

## Supplementary Material

Supplementary Data
