# Supplementary material for: DisVis: quantifying and visualizing accessible interaction space of distance-restrained biomolecular complexes
Source: Bioinformatics. 2015 May 29;31(19):3222–4. doi: 10.1093/bioinformatics/btv333 (PMC4576694; doi:10.1093/bioinformatics/btv333)
Supplement: Supplementary Data [file supp_31_19_3222__index.html]

DisVis: Quantifying and visualizing accessible interaction space of distance-restrained biomolecular complexes — DisVis: quantifying and visualizing accessible interaction space of distance-restrained biomolecular complexes — DisVis: quantifying and visualizing accessible interaction space of distance-restrained biomolecular complexes — Supplementary Data 

# DisVis: quantifying and visualizing accessible interaction space of distance-restrained biomolecular complexes

## Supplementary Data

files

- Supplementary Data - pdf file
