# Supplementary material for: DisVis: quantifying and visualizing accessible interaction space of distance-restrained biomolecular complexes
Source: Bioinformatics. 2015 May 29;31(19):3222–4. doi: 10.1093/bioinformatics/btv333 (PMC4576694; doi:10.1093/bioinformatics/btv333)
Supplement: Supplementary Data [file supp_btv333_DisVis-supplementary_information.pdf]

Supplementary Information

DisVis: Quantifying and visualizing accessible interaction space of distance-restrained biomolecular complexes

G.C.P. van Zundert[1] and A.M.J.J. Bonvin[1]

[1]Bijvoet Center for Biomolecular Research, Faculty of Science - Chemistry, Utrecht University, Utrecht, 3584CH, the Netherlands

1 Calculating the accessible interaction space of two interacting macromolecules

As a first approximation to calculate the accessible interaction space of two interacting macromolecules and to make the computation more tractable, we treat the molecules as rigid entities. This results in a 6 dimensional (3 translational and 3 rotational degrees of freedom) space of possible conformations that need to be considered. To determine within this 6D space whether the two chains are interacting and forming a complex, we use Fast Fourier Transform (FFT)-techniques as used originally in Katchalski-Katzir *et al.* (1992). We keep one chain fixed during the search while we perform FFT-accelerated translational scans with the other chain. The fixed chain is separated into a core and interaction region. The core region is the space that is occupied by combining spheres with each center at the atom coordinate and as radius the elements' van der Waals radius; the interaction region is determined similarly, but the radius is extended by 3Å (by default). The 3D shapes are subsequently projected onto a grid with a voxel spacing of 1Å (by default). The scanning chain is only represented by its core object. The resulting shape is again projected onto a grid with equal voxel spacing as the fixed chain to allow for FFT-accelerated translational scans during the search.

After the creation of the search objects, we identify clashes and interactions as a function of rotation R as follows

$$\boldsymbol{C}(R) = \mathcal{F}^{-1}[\mathcal{F}(\boldsymbol{S}(R))^{*} \times \mathcal{F}(\boldsymbol{F}_{\mathrm{core}})] \qquad \text{(Eq. 1)}$$
$$\boldsymbol{I}(R) = \mathcal{F}^{-1}[\mathcal{F}(\boldsymbol{S}(R))^{*} \times \mathcal{F}(\boldsymbol{F}_{\mathrm{inter}})] \qquad \text{(Eq. 2)}$$

where the cross-correlation theorem has been used to calculate $\boldsymbol{C}$ and $\boldsymbol{I}$, the spaces that represent the volume of clashes and interactions at every grid position in Å^3, respectively; $\mathcal{F}$ and $\mathcal{F}^{-1}$ represent the Fast Fourier Transform operator and its inverse, respectively; * is the complex conjugate operator, and $\times$ the elementwise multiplication operator; $\boldsymbol{S}$ is the shape of the scanning chain, and $\boldsymbol{F}_{\mathrm{core}}$ and $\boldsymbol{F}_{\mathrm{inter}}$ are the core and interaction shapes of the fixed chain, respectively.

To determine whether a conformation is a plausible complex its clashing volume should not be too large, while the interaction volume should be of reasonable size. The accessible interaction space per translational space is then given by

$$\boldsymbol{A}_R(\vec{\boldsymbol{r}}) = \begin{cases} \text{if } \boldsymbol{C}_R(\vec{\boldsymbol{r}}) \leq C_{\max} \text{ and } \boldsymbol{I}_R(\vec{\boldsymbol{r}}) \geq I_{\min}: 1 \\ \text{else} : 0 \end{cases} \qquad \text{(Eq. 3)}$$

where $C_{\max}$ and $I_{\min}$ are parameters representing the allowed maximum volume of clashes (200Å^3 by default) and the minimum volume of interactions (300A^3 by default), respectively. Raising $C_{\max}$ and lowering $I_{\min}$ results in a more lenient counting of accessible states, while lowering $C_{\max}$ and raising $I_{\min}$ makes the counting for accessible states more stringent. The total number of accessible states is determined by performing an

exhaustive search over rotation space and counting at every rotation all states where $\boldsymbol{A}_R$ equals 1. Care should be taken here that rotation space is as evenly and optimally sampled as possible to minimize redundancy and biasing certain orientations in the counting. To take this into account, we used the optimal rotation sets developed by Karney (2007), which include a weight factor for every rotation to average out redundancy. The total number of accessible states N_A is thus given by

$$N_A = \sum_{\mathbf{P}} w_R \sum_{x,y,z} \boldsymbol{A}_R(x, y, z) \qquad \text{(Eq. 4)}$$

where w_R is the weight factor for the specific orientation/rotation, the first summation is over all rotations $\mathbf{P}$, and the second summation over all grid coordinates.

2 Incorporating distance restraints into the search

As mentioned in the main text, if some distances or distance ranges are known between the subunits of the complex, this can significantly reduce the accessible interaction space as this puts extra restraints on the requirement for a conformation to be considered a complex. To combine this information with FFT-accelerated translational scans, the whole space of conformations that comply with the distance restraint should be demarcated for every rotation at once. As the distance of the restraint depends only on the coordinates of two atoms (or points, more generally), the space consistent with the restraint must be represented by a sphere with a radius corresponding to the distance restraint. The remaining parameter that needs to be determined is the position of the center of this sphere $\vec{\boldsymbol{r}}_c$, which is given by

$$\vec{\boldsymbol{r}}_c(R) = \vec{\boldsymbol{r}}_{\mathrm{F}} - (\vec{\boldsymbol{r}}_{\mathrm{S}}(R) - \vec{\boldsymbol{r}}_{\mathrm{comS}}(R)) \qquad \text{(Eq. 5)}$$

where $\vec{\boldsymbol{r}}_{\mathrm{F}}$ and $\vec{\boldsymbol{r}}_{\mathrm{S}}$ are the coordinates of the restrained atoms of the fixed and scanning chain, respectively, and $\vec{\boldsymbol{r}}_{\mathrm{comS}}$ is the center of mass of the scanning chain. The equation can be simplified by initially placing the center of mass of the scanning chain on the origin, and rotating the scanning chain around its center of mass. Furthermore, realizing that $\vec{\boldsymbol{r}}_{\mathrm{F}}$ is fixed and $\vec{\boldsymbol{r}}_{\mathrm{S}}$ now only depends on the rotation of the scanning chain, Eq. 5 reduces to

$$\vec{\boldsymbol{r}}_c(R) = \vec{\boldsymbol{r}}_{\mathrm{F}} - R\,\vec{\boldsymbol{r}}_{\mathrm{S}} \qquad \text{(Eq. 6)}$$

The space of states complying with the distance restraint per translational scan $\boldsymbol{L}_R$ is defined then as

$$\boldsymbol{L}_R(\vec{\boldsymbol{r}}) = \begin{cases} \text{if } d_{\min} \le |\vec{\boldsymbol{r}} - \vec{\boldsymbol{r}}_c| \le d_{\max} : 1 \\ \text{else} : 0 \end{cases} \qquad \text{(Eq. 7)}$$

where $d_{\min}$ and $d_{\max}$ are the minimum and maximum allowed distance, respectively. Note that the function describing $\boldsymbol{L}_R$ can be freely chosen, under the restriction that it should be spherical symmetric, which opens up the use of more complex distance restraints in FFT-docking software. The reduced accessible interaction space $\boldsymbol{A}_{R,\,\mathrm{red.}}$ is then simply given by

$$\boldsymbol{A}_{R,\,\mathrm{red.}} = \boldsymbol{A}_R \times \boldsymbol{L}_R \qquad \text{(Eq. 8)}$$

In the case of multiple available distance restraints, this generalizes to

$$\boldsymbol{A}_{R,\,\mathrm{red.}} = \boldsymbol{A}_R \times \sum_n^{N_d} \boldsymbol{L}_{R,n} \qquad \text{(Eq. 9)}$$

where the summation is over all distance restraints N_d and $\boldsymbol{L}_{R,n}$ is the space conforming to distant restraint n. The value found at a specific coordinate in $\boldsymbol{A}_{R,\text{red.}}$ represents the number of conforming distance restraints at that location in space. The restraints are defined in a separate text file as input for *disvis* in the following format

```
<chain-1> <resi-1> <atomname-1> <chain-2> <resi-2> <atomname-2> <min-dis> <max-dis>
```

where <chain-1>, <resi-1> and <atomname-1> are the chainID, residue number, and atom name of the fixed chain, respectively; <chain-2>, <resi-2> and <atomname-2> are the chainID, residue number, and atom name of the scanning chain; and <min-dis> and<max-dis> are the minimal and maximal distance of the restraints in angstrom, respectively. As an example, the line

```
A 18 CA F 27 CB 10.0 20.0
```

would put a restraint between the CA-atom of residue 18 of chain A of the fixed model and the CB-atom of residue 27 of the F chain of the scanning model that should be longer of equal to 10Å and shorter or equal to 20Å.

3 Quantifying and visualizing the accessible interaction space

To quantify the accessible interaction space consistent with a certain number of distance restraints, the number of occurrences that $\boldsymbol{A}_{R,\text{red.}}$ is equal to the number of compliant restraints is counted. The accessible interaction space is visualized by outputting the maximum value found during the rotational search at every grid position, thus given by

$$\boldsymbol{V}(x,y,z) = \max\{\boldsymbol{A}_{R,\text{red.}}(x,y,z) : R \in \mathbf{P}\} \qquad \text{(Eq. 10)}$$

The resulting 'density' is written to file in MRC format and represents the position of the center of mass of the scanning chain relative to the fixed chain. These files can straightforwardly opened with molecular visualization programs, such as PyMol and UCSF Chimera. With this information, interesting regions of high-density can then be sampled more thoroughly. Also, false-positive restraints can be identified if the exhaustive search does not result in a region where all restraints of the cross-links are obeyed. In addition, for each complex that is consistent with at least one restraint, all restraints that are violated are calculated and stored during the search. This ultimately results in a violation matrix where every row represents the number of consistent restraints N and every column indicates how often a specific restraint is violated for complexes consistent with at least N restraints (e.g. Table S3). Lastly, to give the user an indication which restraints are most likely to be false-positives, the z-score is calculated for each restraint based on the violation matrix given by

$$z = \frac{v_i - \bar{v}}{\sigma} \qquad \text{(Eq. 11)}$$

where v_i is the column average of the violation matrix of restraint i, and $\bar{v}$ and σ are the average and standard deviation of the violation matrix. *Disvis* reports restraints with a z-score higher than 1.0 explicitly.

4 Output files of *disvis*

Below we describe the 5 output files of *disvis* after a run together with example output of the RNA polymerase II example from the main text.

- *accessible_complexes.out*: a text file containing the number of complexes consistent with a given number of restraints. Column 1 shows the number of consistent restraints for each complex counted, denoted by N; column 2 shows the number of complexes consistent with *exactly* N restraints; column 3 shows the fraction of all complexes sampled that are consistent with *exactly* N restraints; column 4 gives the number of complexes consistent with *at least* N restraints, and is thus the cumulative sum of

column 2; column 5 is again the fraction of complexes consistent with *at least* N restraints, and also the cumulative sum of column 3.

For example the following output file shows that (row 1) more than 18.9×10^9 complexes were sampled of which 16.6×10^9 (87.5%) are consistent with *exactly* zero restraints, i.e. all restraints are violated by these complexes. Of those 18.9×10^9 complexes, 2.4×10^9 were consistent with *at least* 1 restraint and 1.4×10^9 consistent with *exactly* 1 restraint. Furthermore, no complexes were found that were consistent with all 8 available restraints, indicating the presence of at least one false-positive.

```
 0  16570457037 0.874857  18940752204 1.000000
 1   1392884181 0.073539   2370295166 0.125143
 2    678488947 0.035822    977410985 0.051604
 3    206270378 0.010890    298922038 0.015782
 4     74963882 0.003958     92651659 0.004892
 5     12515339 0.000661     17687776 0.000934
 6      5162720 0.000273      5172437 0.000273
 7         9716 0.000001         9716 0.000001
 8            0 0.000000            0 0.000000
```

- *violations.out*: a text file showing how often a specific restraint is violated for complexes consistent with a number of restraints. The higher the violation fraction of a specific restraint, the more likely it is to be a false-positive. Column 1 shows the number of consistent restraints N, while each following column indicates the violation fractions of a specific restraint for complexes consistent with *at least* N restraints. Each row thus represents the fraction of all complexes consistent with *at least* N restraints that violated a particular restraint.

  Again, we will go through an example of interpreting the data. It is most informative here to start from the bottom of the table. Since no complexes were found that are consistent with all 8 restraints, the violation fraction in row 8 are all set to 0. Row 7 shows that for complexes consistent with *at least* 7 restraints, it is clear that restraint 8 is violated by all of them (100%), thus indicating that it is a false-positive, which indeed was the case. Next, row 6 shows that for all complexes that were consistent with *at least* 6 restraints, restraint 7 is violated in 99.65% of them and restraint 8 still in all (100%) of them. Thus, from these data we can extract that restraint 7 and 8 are most likely the false-positives.

```
          1       2       3       4       5       6       7       8
1    0.7307  0.8134  0.7813  0.8129  0.7417  0.7798  0.7718  0.9812
2    0.6764  0.6174  0.5856  0.7254  0.5037  0.4972  0.9739  0.9964
3    0.3079  0.3441  0.2854  0.4343  0.6538  0.6221  0.9698  0.9961
4    0.0795  0.1509  0.0565  0.2383  0.6531  0.6070  0.9681  0.9998
5    0.0150  0.1396  0.0011  0.3714  0.1795  0.0609  0.9397  0.9999
6    0.0004  0.0000  0.0000  0.0001  0.0011  0.0000  0.9965  1.0000
7    0.0000  0.0000  0.0000  0.0000  0.0000  0.0000  0.0000  1.0000
8    0.0000  0.0000  0.0000  0.0000  0.0000  0.0000  0.0000  0.0000
```

- *accessible_interaction_space.mrc*: a density file in MRC format. The density represents the center of mass of the scanning chain conforming to the maximum found consistent restraints at every position in space. The density can be inspected by opening it together with the fixed chain in a molecular viewer (UCSF Chimera is recommended for its easier manipulation of density data, but also PyMol works). See Figure 1 and Figure S1 for an example.

- *z-score.out*: a text file giving the Z-score for each restraint. The higher the score, the more likely the restraint is a false-positive. Z-scores above 1.0 are explicitly mentioned in the output. The first column indicates the restraint; column 2 gives the average violation fraction, i.e. the average of the corresponding column in *violations.out*; column 3 represents the standard deviation of the average violation fraction; and column 4 finally gives the z-score.

The following file shows that restraints 7 and 8 have the highest violation faction, and their z-score is both higher than 1. *Disvis* will thus indicate them as possible false-positives.

```
1  0.259  0.299 -0.795
2  0.295  0.291 -0.655
3  0.244  0.298 -0.850
4  0.369  0.297 -0.371
5  0.390  0.298 -0.288
6  0.367  0.310 -0.379
7  0.803  0.335  1.298
8  0.996  0.006  2.041
```

- *disvis.log*: a log file showing all the parameters used, together with date and time indications.

5 Additional implementation details

We implemented DisVis in Python2.7 using the NumPy (Van der Walt *et al*., 2011) and Cython packages (Behnel *et al*., 2011). The OpenCL framework (Stone *et al*., 2010) was used to offload the computations to the GPU. Python bindings were available through the pyopencl package (Klöckner *et al.,* 2012). We used the high-performance clFFT library (https://github.com/clMathLibraries/clFFT) together with gpyfft for Python bindings (https://github.com/geggo/gpyfft) to calculate the FFT's. Computations were performed on AMD Opteron 6344 CPU processors and on an AMD Radeon HD 7730M and NVIDIA GeForce GTX 680 GPU.

6 RNA polymerase II example

The crystal structure of the RNA polymerase II was downloaded from the Protein Databank (PDB ID: 1WCM). The largest subunit (chain A) and the 27kDa polypeptide (chain E) were extracted from the PDB-file. Six BS3 cross-links were available and taken from XLdb (Kahraman *et al*., 2013). To investigate the detection of false-positive restraints, two virtual cross-links were added with a distance of 35.7 and 42.2Å using the Xwalk webserver (Kahraman *et al*. 2011). The maximum allowed distance of the BS3 cross-links was set to 30Å, based on molecular dynamics trajectory analysis (Merkley *et al*., 2014). The restraints used are shown in Table S1. The input files are included in the DisVis source code.
Two *disvis* runs were performed using a rotational sampling density of 5.27° (53256 orientations) and 9.72° (7416 orientations) with a grid spacing of 1Å and 2Å, respectively. All parameters were left to there default values (interaction radius 3Å, minimum required volume of interaction 300Å^3 and maximum allowed volume of clashes 200Å^3). The amount of accessible complexes consistent with each number of cross-links is shown in Table S2 and S4, and the relative occurrence of restraint violations in Table S3 and S5 for the fine and coarse run, respectively.

7 26S proteasome PRE5-PUP2 example

Homology models were downloaded from the SWIS-MODEL Repository (Kiefer *et al*., 2009) via the Protein Model Portal (http://proteinmodelportal.org) using their Uniprot identifiers (O14250 and Q9UT97). Cross-links were taken from Leitner *et al*. (2014) Dataset S1 (Table S6), which consist of 4 adipic acid dihydrazide (ADH) and 3 zero-length (ZL) cross-links. The maximum ADH- and ZL linker length were set to 23Å and 26Å, respectively, since 95% of all found distances in a benchmark were smaller. All input files are included in the DisVis source code.
Again, two *disvis* runs were performed using a rotational sampling density of 5.27° (53256 orientations) and 9.72° (7416 orientations) with a grid spacing of 1Å and 2Å, respectively, with default parameter values. The sum of accessible complexes consistent with each number of restraints is shown in Table S7 and S9, and their normalized restraint violation occurrence in Table S8 and S10.

Supplementary Tables

Table S1. Cross-links used to assess accessible interaction space of the large subunit and the 27kDa polypeptide of the RNA polymerase II complex.

Residue chain A	Residue chain E	Cross-linker[†]	Distance in complex (Å)[‡]
1003	166	BS3	12.5
129	161	BS3	19.8
129	171	BS3	12.9
15	171	BS3	19.6
934	201	BS3	21.8
938	201	BS3	15.1
180	122	Virtual	35.7
1092	152	Virtual	42.2

[†]Cross-link chemistry. BS3: Bissulfosuccinimidyl suberate; Virtual: Manually added false-positive cross-link.

[‡] C_β- C_β distance in crystal structure (1WCM)

Table S2. Total number of accessible complex conformations per number of complying restraints of the large subunit and the 27kDa polypeptide of the RNA polymerase II complex using a fine rotational search (5.27°, 53256 orientations) and grid (1Å).

Number of consistent restraints (N)	Number of accessible complexes consistent with exactly N restraints	Fraction of accessible complexes consistent with exactly N restraints	Number of accessible complexes consistent with at least N restraints	Fraction of accessible complexes consistent with at least N restraints
0	16570457037	0.8749	18940752204	1.0000
1	1392884181	0.0735	2370295166	0.1251
2	678488947	0.0358	977410985	0.0516
3	206270378	0.0109	298922038	0.0158
4	74963882	0.0040	92651659	0.0049
5	12515339	0.0007	17687776	0.0009
6	5162720	0.0003	5172437	0.0003
7	9716	0.0000	9716	0.0000
8	0	0.0000	0	0.0000

Table S3. Normalized occurrence of a restraint violation given a number of consistent restraints for the large subunit and the 27kDa polypeptide of the RNA polymerase II complex using a fine rotational search (5.27°, 53256 orientations) and grid (1Å).

Number of consistent restraints (N)	Percentage of complexes consistent with a given number of restraints[a] in which a specific restraint is violated							
	Restraint 1	Restraint 2	Restraint 3	Restraint 4	Restraint 5	Restraint 6	Restraint 7	Restraint 8
1	0.731	0.813	0.781	0.813	0.742	0.780	0.772	0.981
2	0.676	0.617	0.586	0.725	0.504	0.497	0.974	0.996
3	0.308	0.344	0.285	0.434	0.654	0.622	0.970	0.996
4	0.080	0.151	0.057	0.238	0.653	0.607	0.968	1.000
5	0.015	0.140	0.001	0.371	0.180	0.061	0.940	1.000
6	0.000	0.000	0.000	0.000	0.001	0.000	0.997	1.000
7	0.000	0.000	0.000	0.000	0.000	0.000	0.000	1.000
8[b]	0.000	0.000	0.000	0.000	0.000	0.000	0.000	0.000

a) This is the number of conformations reported in Table S2 column 4

b) All values for 8 restraints are 0 because there are no single conformation satisfying all 8 restraints (see last row of Table S2)

Table S4. Total number of accessible complex conformations per number of complying restraints of the large subunit and the 27kDa polypeptide of the RNA polymerase II complex using a coarse rotational search (9.72°, 7416 orientations) and grid (2Å).

Number of consistent restraints (N)	Number of accessible complexes consistent with exactly N restraints	Fraction of accessible complexes consistent with exactly N restraints	Number of accessible complexes consistent with at least N restraints	Fraction of accessible complexes consistent with at least N restraints
0	287850752	0.8757	328691520	1.0000
1	24045812	0.0732	40840780	0.1243
2	11681382	0.0355	16794968	0.0511
3	3538009	0.0108	5113586	0.0156
4	1281164	0.0039	1575577	0.0048
5	208446	0.0006	294412	0.0009
6	85798	0.0003	85966	0.0003
7	167	0.0000	167	0.0000
8	0	0.0000	0	0.0000

Table S5. Normalized occurrence of a restraint violation given a number of consistent restraints for the large subunit and the 27kDa polypeptide of the RNA polymerase II complex using a coarse rotational search (9.72°, 7416 orientations) and grid (2Å).

Number of consistent restraints (N)	Percentage of complexes consistent with a given number of restraints[a] in which a specific restraint is violated							
	Restraint 1	Restraint 2	Restraint 3	Restraint 4	Restraint 5	Restraint 6	Restraint 7	Restraint 8
1	0.731	0.812	0.780	0.811	0.743	0.782	0.774	0.981
2	0.679	0.615	0.583	0.724	0.507	0.501	0.974	0.997
3	0.313	0.339	0.282	0.431	0.659	0.628	0.970	0.996
4	0.080	0.146	0.055	0.235	0.660	0.615	0.967	1.000
5	0.015	0.135	0.001	0.373	0.182	0.063	0.937	1.000
6	0.000	0.000	0.000	0.000	0.001	0.000	0.996	1.000
7	0.000	0.000	0.000	0.000	0.000	0.000	0.000	1.000
8	0.000	0.000	0.000	0.000	0.000	0.000	0.000	0.000

a) This is the number of conformations reported in Table S4 column 4

Table S6. Cross-links used to assess the accessible interaction space of PUP2 relative to PRE5. Data were taken from Leitner *et al*. (2014) Dataset S1.

Residue PRE5	Residue PUP2	Cross-linker[†]	Distance in complex (Å)[‡]
27	18	ADH	5.9
122	125	ADH	12.1
122	127	ADH	5.7
122	128	ADH	7.8
54	179	ZL	9.1
55	169	ZL	10.8
55	179	ZL	10.9

[†]Cross-link chemistry. ADH: adipic acid dihydrazide; ZL: zero-length

[‡] C_α- C_α distance in homology model

Table S7. Total number of accessible complex conformations per number of complying restraints of the PRE5-PUP2 complex using a fine rotational search (5.27°, 53256 orientations) and grid (1Å).

Number of consistent restraints (N)	Number of accessible complexes consistent with exactly N restraints	Fraction of accessible complexes consistent with exactly N restraints	Number of accessible complexes consistent with at least N restraints	Fraction of accessible complexes consistent with at least N restraints
0	5431316957	0.7837	6930088505	1.0000
1	565217635	0.0816	1498771547	0.2163
2	226110049	0.0326	933553912	0.1347
3	622583287	0.0898	707443862	0.1021
4	73552113	0.0106	84860574	0.0122
5	4747627	0.0007	11308461	0.0016
6	4069363	0.0006	6560833	0.0009
7	2491469	0.0004	2491469	0.0004

Table S8. Normalized occurrence of a restraint violation given a number of consistent restraints for the PRE5-PUP2 complex using a fine rotational search (5.27°, 53256 orientations) and grid (1Å).

Number of consistent restraints (N)	Percentage of complexes consistent with a given number of restraints[a] in which a specific restraint is violated						
	Restraint 1	Restraint 2	Restraint 3	Restraint 4	Restraint 5	Restraint 6	Restraint 7
1	0.717	0.739	0.726	0.729	0.620	0.648	0.656
2	0.863	0.641	0.596	0.576	0.507	0.465	0.483
3	0.855	0.585	0.569	0.572	0.431	0.421	0.418
4	0.077	0.481	0.442	0.456	0.450	0.438	0.417
5	0.319	0.346	0.055	0.137	0.211	0.110	0.023
6	0.227	0.232	0.003	0.005	0.121	0.033	0.000
7	0.000	0.000	0.000	0.000	0.000	0.000	0.000

a) This is the number of conformations reported in Table S7 column 4.

Table S9. Total number of accessible complex conformations per number of complying restraints of the PRE5-PUP2 complex using a coarse rotational search (9.72°, 7416 orientations) and grid (2Å).

Number of consistent restraints (N)	Number of accessible complexes consistent with exactly N restraints	Fraction of accessible complexes consistent with exactly N restraints	Number of accessible complexes consistent with at least N restraints	Fraction of accessible complexes consistent with at least N restraints
0	96048647	0.7871	122031044	1.0000
1	9884350	0.0810	25982397	0.2129
2	3940305	0.0323	16098046	0.1319
3	10681417	0.0875	12157741	0.0996
4	1281763	0.0105	1476323	0.0121
5	81860	0.0007	194559	0.0016
6	70057	0.0006	112699	0.0009
7	42641	0.0003	42641	0.0003

Table S10. Normalized occurrence of a restraint violation given a number of consistent restraints for the PRE5-PUP2 complex using a coarse rotational search (9.72°, 7416 orientations) and grid (2Å).

Number of consistent restraints (N)	Percentage of complexes consistent with a given number of restraints[a] in which a specific restraint is violated						
	Restraint 1	Restraint 2	Restraint 3	Restraint 4	Restraint 5	Restraint 6	Restraint 7
1	0.713	0.736	0.724	0.726	0.628	0.654	0.662
2	0.861	0.635	0.590	0.570	0.515	0.471	0.489
3	0.853	0.578	0.561	0.565	0.439	0.428	0.426
4	0.076	0.476	0.438	0.452	0.457	0.443	0.422
5	0.320	0.340	0.056	0.136	0.218	0.110	0.022
6	0.227	0.229	0.004	0.005	0.125	0.032	0.000
7	0.000	0.000	0.000	0.000	0.000	0.000	0.000

a) This is the number of conformations reported in Table S9 column 4

Table S11. Profiling *disvis* for a 20.83° rotational search (648 orientations) using a 1Å grid spacing of RNA polymerase II large subunit and 27kDa polypeptide

Function	Time (s)	Percentage of total (%)
Determining consistent distance restraint space	7	1%
Flattening arrays (method 'flatten' of 'numpy.ndarray' objects)	8	1%
Filling arrays (method 'fill' of 'numpy.ndarray' objects)	9	1%
Counting violations	11	2%
Reduce (method 'reduce' of 'numpy.ufunc' objects)	12	2%
Complex conjugate (method 'conj' of 'numpy.ndarray' objects)	19	3%
Rotating the scanning chain	24	3%
Copying of arrays (method 'copy' of 'numpy.ndarray' objects)	36	5%
Binning the number of accessible complexes	37	5%
Main loop (multiplications, summations, etc.)	104	15%
FFT calculations	445	62%
Total time	717	100%

Supplementary Figures

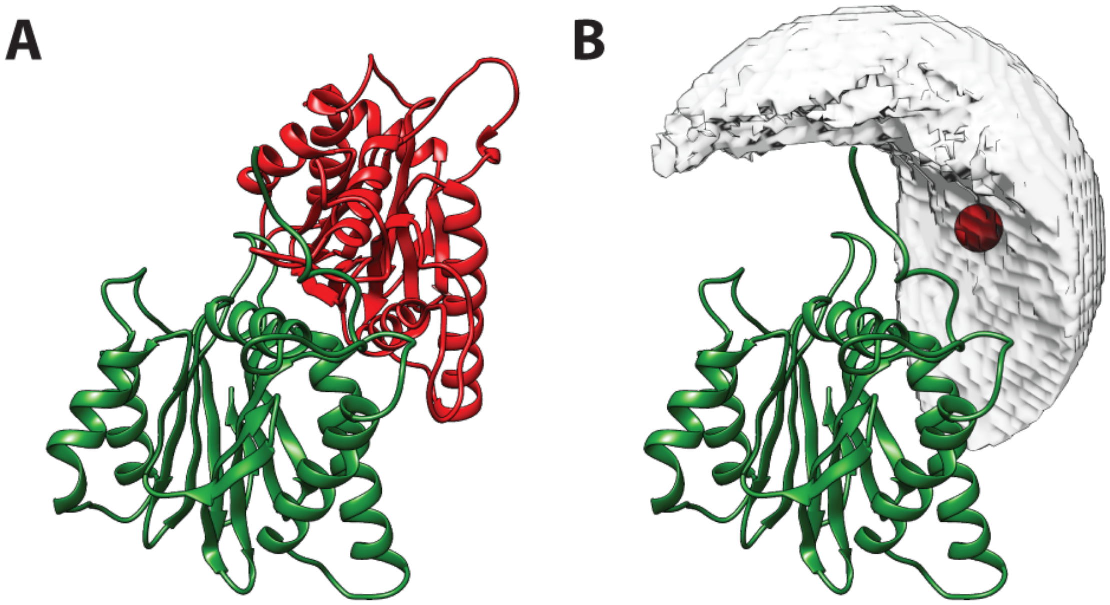

Figure S1. (A) The PRE5 (green) complexed with PUP2 (green), based on a homology model. (B) PRE5 and the accessible interaction space of PUP2 consistent with all 7 distance restraints (grey). The smooth red sphere represents the center of mass of PUP2.

Supplementary References

Behnel, S., Bradshaw, R., Citro, C., Dalcin, L., Seljebotn, D.S. and Smith, K. (2011) Cython: the best of both worlds. *Computing in Science & Engineering,* **13**, 31-39.

Leitner, A., Joachimiak, L.A., Unverdorben, P., Walzthoeni, T., Frydman, J., Förster, F. and Aebersold, R. (2014) Chemical cross-linking/mass spectrometry targeting acidic residues in proteins and protein complexes. *Proc. Natl. Acad. Sci. USA*, **111,** 9455-9460.

Kahraman, A., Malmström, L. and Aebersold, R. (2011) Xwalk: computing and visualizing distances in cross-linking experiments. *Bioinformatics*, **27,** 2163-2164.

Kahraman, A., Herzog, F., Leitner, A., Rosenberger, G., Aebersold, R. and Malmström, L. (2013) Cross-link guided molecular modeling with ROSETTA. *PLoS One*, **8**, e73411.

Karney, C.F.F. (2007) Quaternions in molecular modeling. *J. Mol. Graph. Model*, **25**, 595-604.

Kiefer, F., Arnold, K., Künzli, M., Bordoli, L. and Schwede, T. (2009) The SWISS-MODEL Repository and associated resources. *Nucleic Acids Res.,* **37**, D387-D392.

Klöckner, A., Pinto, N., Lee, Y., Catanzaro, B., Ivanov, P. and Ahmed, F. (2012) PyCUDA and PyOpenCL: A scripting-based approach to GPU run-time. *Parallel Computing*, **38,** 157-174.

Merkley, E.D., Rysavy, S., Kahraman, A., Hafen, R.P., Daggett, V. and Adkins, J.N. (2014) Distance restraints from crosslinking mass spectrometry: mining a molecular dynamics simulation database to evaluate lysine-lysine distances. *Protein Sci.,* **23**, 747-759.

Stone, J.E., Gohara, D. and Shi, G. (2010) OpenCL: a parallel programming standard for heterogeneous computing systems. *IEEE Des. Test*, **12**, 66-73.

Van der Walt, S., Colbert, S.C. and Varoquaux, G. (2011) The NumPy array: a structure for efficient numerical computation. *Computing in Science & Engineering,* **13**, 22-30.
